# Identification of *Listeria monocytogenes* Contamination in a Ready-to-Eat Meat Processing Plant in China

**DOI:** 10.3389/fmicb.2021.628204

**Published:** 2021-02-25

**Authors:** Hongzhi Zhang, Fengxia Que, Biyao Xu, Linjun Sun, Yanqi Zhu, Wenjie Chen, Yulong Ye, Qingli Dong, Hong Liu, Xi Zhang

**Affiliations:** ^1^Shanghai Municipal Center for Disease Control and Prevention, Shanghai, China; ^2^The Jinshan District Center for Disease Control and Prevention, Shanghai, China; ^3^Institute of Food Quality and Safety, University of Shanghai for Science and Technology, Shanghai, China

**Keywords:** *Listeria monocytogenes*, RTE meat processing plant, PFGE, WGS, cgMLST

## Abstract

*Listeria monocytogenes* is the etiologic agent of listeriosis, which remains a significant public health concern in many countries due to its high case-fatality rate. The constant risk of *L. monocytogenes* transmission to consumers remains a central challenge in the food production industry. At present, there is very little known about *L. monocytogenes* contamination in ready-to-eat (RTE) processing plants in China. In this study, *L. monocytogenes* in an RTE meat processing plant in Shanghai municipality was characterized using pulsed-field gel electrophoresis (PFGE) and whole genome sequencing (WGS). Furthermore, the biofilm formation ability of the pathogen was also tested. Results revealed that *L. monocytogenes* isolates were present in 12 samples out of the 48 samples investigated. Most of them (66.7%, 8/12) were identified from the processing facilities irrespective of observed hygiene levels of aerobic plate count (APC) and coliforms. Coliforms were present in only one processing area. ST5 (1/2b) isolates were predominant (83.3%, 10/12) and were identified in two dominant pulsotypes (PTs) (three in PT3 and seven in PT4, respectively). Results of the core-genome multi-locus sequence typing (cgMLST) showed that ST5 in three PTs (PT1, PT3, and PT4) had 0–8 alleles, which confirmed that clonal transmission occurred in the RTE meat processing facilities. In addition, the biofilm formation test confirmed that the isolates from the processing facilities could form biofilms, which helped them colonize and facilitate persistence in the environment. These results indicated that common sanitation procedures regularly applied in the processing environment were efficient but not sufficient to remove *L. monocytogenes* isolates, especially biofilm of *L. monocytogenes*. Furthermore, the ST5 isolates in this study exhibited 12 alleles with one ST5 clinical isolate, which contributes to the understanding of the potential pathogenic risk that *L. monocytogenes* in RTE meat processing equipment posed to consumers. Therefore, strong hygienic measures, especially sanitation procedures for biofilms eradication, should be implemented to ensure the safety of raw materials. Meanwhile, continuous surveillance might be vital for the prevention and control of listeriosis caused by *L. monocytogenes*.

## Introduction

*Listeria monocytogenes* is an important foodborne pathogen, which can cause severe human listeriosis, particularly in older adults, newborns, pregnant women, and immune-compromised individuals (Lomonaco et al., 2009). Listeriosis remains a significant public health concern due to the high case-fatality rate (Thomas et al., 2015). Various types of meat, especially ready-to-eat (RTE) meat products, are often vehicles of listeriosis outbreaks (Currie et al., 2015; Jensen et al., 2016; Thomas et al., 2020). A risk assessment report from the United States in 2003 attributed 90% of listeriosis cases to the consumption of contaminated RTE deli meats (Richard Whiting, 2003). In the United States, *L. monocytogenes* must not present in RTE foods at any point (USDA FSIS, 2003). A similar requirement in China is that *L. monocytogenes* is not detectable in 25 g or 25 ml RTE food according to GB29921 (GB29921-2013,2013).

*L. monocytogenes* survives well in the environment and can even colonize food production facilities for extended periods (Leong et al., 2017). *L. monocytogenes* is known to colonize niche areas such as drains and hard-to-clean surfaces, which allows the bacteria to survive or even proliferate and thus make it challenging to completely eradicate it (Ferreira et al., 2014). An epidemiological investigation of listeriosis outbreaks revealed significant lack of hygiene in processing facilities (Angelo et al., 2017). Several reported outbreaks caused by *L. monocytogenes* have been linked to contaminated food-contact surfaces, packing lines, and processing environments (McCollum et al., 2013; Angelo et al., 2017). A high prevalence of *L. monocytogenes* in food processing environments is often reported. However, a smaller number of studies have evaluated the incidence and identified the potential of *L. monocytogenes* contamination in RTE meat processing plants (Nastasijevic et al., 2017). The risk of *L. monocytogenes* transmission to consumers remains a central challenge for the food industry (Almeida et al., 2013; Malley et al., 2015).

Molecular typing of *L. monocytogenes* isolates can help to establish links between isolates from different sources and assist in tracing the original source of contamination (Chen et al., 2013). It has been reported that certain serotypes and clonal complexes (CCs) are more commonly encountered in clinical cases (Chenal-Francisque et al., 2011; Maury et al., 2016). In China, ST87 and ST8 were the most prevalent types of isolates from patients (Li et al., 2019). However, ST9 was the most common type of isolates from foods (Wang et al., 2012).

Pulsed-field gel electrophoresis (PFGE), as the “gold standard” typing method, has been used to characterize clusters of *L. monocytogenes* isolates through the National Molecular Tracing Network for Foodborne Disease Surveillance (TraNet) in China (Li et al., 2019). Whole genome sequencing (WGS) is a powerful tool for obtaining genomic data, which can help to determine sequence types (STs), serogroups, virulence, and resistance gene profiles (Li et al., 2017). More importantly, several molecular typing methods have been developed using WGS data such as core-genome multiple locus sequence typing (cgMLST) and single nucleotide polymorphism (SNP). Recently, WGS has been used to determine the contamination and/or colonization routes of pathogens within food processing environments (Food and Agriculture Organization of the United Nations, 2016; Wang et al., 2016). The aim of this study was (1) to identify the transmission routes of *L. monocytogenes* using PFGE and WGS via tracking *L. monocytogenes* isolates in an RTE meat processing plant in Shanghai, as well as (2) to provide a basis for measures to prevent and control the transmission of *L. monocytogenes* in RTE meat processing plants.

## Materials and Methods

### Processing of RTE Meat Products

An RTE cooked meat product processing plant in Shanghai was used to investigate *L. monocytogenes* contamination in RTE-processing environments and products as well as to track its transmission route. The plant is the one of the most important companies producing RTE meat products in Shanghai, and the RTE meat products are common food in Shanghai. The RTE meat products were processed as follows. Firstly, frozen meats were bought from trade companies as raw materials. Secondly, water thawing took place, and meats were pickled in a pickling liquid with many accessory materials including oil, salt, sauce, vinegar, herbs, and spices. Thirdly, the pickled meat products were boiled to produce intermediate products. These intermediate products were eventually processed by activities such as weighing and cutting into shapes to create end products. Finally, end products were packaged and transported to retail stores to be eventually purchased and used by consumers.

### Sampling

As shown in Table 1, a total of 48 samples were collected during one visit in July 2019, including 21 processing environmental samples, 10 processing facility samples, 3 raw materials, 3 accessory materials, 8 intermediate products, and 3 end products. After the cleaning and sanitation procedures were complete, the processed environmental samples and facility samples were collected from associated surfaces using pre-moistened swabs (Table 1). Air samples were collected using Anderson six-stage sampler. The raw materials were frozen meat products bought from trade companies. Intermediate products during the processing stage included pickled products and boiled products. The end products were RTE meat products, which were destined for retail and consumption. Samples of these products were delivered to the laboratory within 2 h, in a cold chain. Here, the levels of aerobic plate count (APC) and coliforms were determined, as well as the presence of *L. monocytogenes*.

**TABLE 1:** Sampling locations in an RTE processing plant and detection of *L. monocytogenes* isolates.

Sampling	Samples source/No. of samples	Samples source/No. of *L. monocytogenes*
Processing environments	Water^*b*^/2; power switches^*b*^/2; doorknobs^*b*^/1; floors^*b*^/3; wall^*b*^/1; worker’s hand and shoes^*b*^/3; mops^*b*^/3; air samples from different rooms^*d,e*^/6	–
Processing facilities	Conveyor apparatus^*c*^/2; cutting boards^*d*^/2; knives^*d*^/1; weighing tools^*d*^/2; inside surface of facilities^*d*^/1; outside surface of facilities^*d*^/1; packing bag^*d*^/1	Cutting boards/2; conveyor apparatus/1; knives/1; weighing tools/2; inside surface of facilities/1; outside surface of facilities/1
Raw materials	Thawing meat products^*a*^/3	–
Accessory materials	Pickling liquid^*a*^/3	Pickling liquid/3
Intermediate products	boiled products^*b*^/5; cooled products^*c*^/3	–
End products	RTE meat products^*d*^/3	RTE meat product/1

**Production unit:^a^thawing room. ^*b*^Thermal processing room. ^*c*^Cooling room. ^*d*^Packaging room. ^*e*^Sterilization room.**

### Microbiological Analysis

Enumeration of APC and coliforms were determined according to the food safety national standards GB4789.2 (2016) and GB4789.3 (2016), respectively. *L. monocytogenes* was determined according to the food safety national standard GB4789.30 (2016). Regarding the standard, environmental swabs and raw and accessory materials were placed into *L. monocytogenes* Broth 1 (LB1) for pre-enrichment at 30°C for 24 h. Afterward, 100 μl LB1 was placed into *L. monocytogenes* Broth 2 (LB2) at 30°C for 24 h. One inoculation loop of LB2 was streaked on Polymyxin Acriflavine Licl Ceftazidime Esculin Mannitol Agar Plate (PALCAM). The isolates were identified as *L. monocytogenes* by standard biochemical tests (catalase; fermentation of dextrose, xylose, rhamnose, and mannitol; β-hemolysis; motility; and gram-staining). The positive control strain used in this study was *L. monocytogenes* ATCC 19114.

### PFGE

PFGE for *L. monocytogenes* was performed with the PulseNet International protocol (Centers for Disease Control and Prevention, 2013). Based on the protocol, *L. monocytogenes* isolates were embedded into agarose plugs. Afterward, slices of the agarose plugs were digested using *Asc*I (Takara, Dalian, China) for 3 h at 37°C. XbaI-digested *Salmonella* Braenderup H9812 DNA was used as a molecular size marker, and electrophoresis was conducted using the CHEF-DRII apparatus (Bio-Rad Laboratories, Hercules, CA, United States). Images were captured using the Gel Doc 2000 system (Bio-Rad) and were converted to TIFF files, which were analyzed by the BioNumerics software (version 7.7 Applied Maths, Kortrijk, Belgium). Finally, clustering was performed using the unweighted pair group method with arithmetic mean (UPGMA).

### WGS

Genomic DNA was extracted using the DNeasy Blood & Tissue Kit (QIAGEN, Germany) according to the manufacturer’s protocol except that the cells were pre-lysed with lysozyme for 30 min at 37°C and the proteinase K treatment was extended to 30 min. A Qubit Fluorometer (Invitrogen, United States) and a NanoDrop Spectrophotometer (Thermo Fisher Scientific, United States) were used to determine the concentration, quality, and integrity of the DNA. Sequencing libraries were generated using the TruSeq DNA Sample Preparation Kit (Illumina, United States). Afterward, genome sequencing was performed using the Illumina Hiseq platform (Illumina). Finally, the reads were trimmed and assembled using the CLC Genomics Workbench v7.0 (CLC Bio, Aarhus, Denmark), and the assembled contigs were exported as FASTA files for further analysis.

Ten ST5 *L. monocytogenes* isolates from foods and three isolates from patients were analyzed using WGS to be compared to 10 isolates in this study.

### Serotypes, MLST, and Pathogenic Island Determination

Serotypes of *L. monocytogenes* isolates were identified using a commercially available *L. monocytogenes* antiserum test kit (Denka Seiken, Tokyo, Japan).

Multi-locus sequence typing (MLST) was defined by the Pasteur scheme (Burall et al., 2011) and based on the sequence analysis of the seven housekeeping genes, which were extracted from WGS data using the BioNumerics software.

The virulence associated genes extracted from WGS data using BioNumerics software were put into the Virulence Factor Database (VFDB) (MOH Key Laboratory of Systems Biology of Pathogen, Institute of Pathogen Biology, Beijing, China)^1^ in order to identify LIPI-1, LIPI-2, LIPI-3, and LIPI-4.

### cgMLST Characterization

cgMLST typing was conducted based on the profile of 1,748 coding loci in the BigsDB Pasteur cgMLST^2^. Cluster analysis was conducted by applying a complete linkage using the BioNumerics software.

### Biofilm Formation

The biofilm formation test was conducted according to Pang and Yuk (2018) with minor modifications. Stainless steel (304, Tull Metals Company, Atlanta, GA) coupons (2 cm × 1 cm × 0.2 cm) were soaked in an acetone solution for 3 h. After being wiped clean, these coupons were soaked in 70% (v/v) ethanol overnight and then rinsed with distilled water. After being air-dried and autoclaved at 121°C for 15 min, the coupons were ready for use.

Biofilm was developed on sterile coupons in tryptic soy broth with yeast extract (TSBYE). Firstly, the *L. monocytogenes* were inoculated into TSBYE and cultured overnight at 26°C. Secondly, the inoculum was washed two times with 0.9% sodium chloride and then added to 5 ml TSBYE at the final concentration of 10^5^ CFU/ml for use on the coupons (one coupon in one tube). Afterward, the inoculated TSBYE samples were incubated at 26°C. After incubation for 24 and 48 h, the coupons were removed and placed into 5 ml 0.9% sodium chloride and vortexed for 10 s. Next, the liquid was discarded, and the coupons were placed into a centrifuge (12,000 rpm) with 10 ml 0.9% sodium chloride and glass beads. Finally, after vortexing for 2 min, the liquid was used to enumerate the biofilm cells.

### Statistical Analysis

Each set of experiments was repeated three times, and its mean values (APC, coliforms) and standard deviation were calculated using the SPSS software (17.0, IBM, United States).

## Results

APC and coliforms testing in various samples revealed the hygiene levels within the RTE meat processing plant. APC levels were the highest in raw and accessory materials, while coliforms levels were the highest in one processing facility (Table 2). The level of APC in air samples from processing rooms was 33 CFU/plate.

**TABLE 2:** Hygiene levels in different production units within the meat establishment.

Samples source	Hygiene level indicators	
	APC	Coliforms
Raw and accessory materials	6.15 × 10^5^ CFU/g	<10 CFU/g
End product	4.6 × 10^5^ CFU/g	3.5 × 10^2^ CFU/g
Processing facility	–	2.4 × 10^3^ CFU/cm^2^
Air samples in processing rooms	33 CFU/plate	–

The *L. monocytogenes* isolate occurrence rate was 25% (12/48) (Table 1). Briefly, eight (8/12, 66.7%) *L. monocytogenes* isolates were detected in the processing equipment, which included nearly all production area that had direct contact with products, e.g., the cutting board, conveyor apparatus, knives, inside and outside surfaces of the equipment, and weighing tools (Table 1). The presence of *L. monocytogenes* was confirmed in all RTE processing facilities irrespective of the observed hygiene levels (Table 2).

**FIGURE 1:**
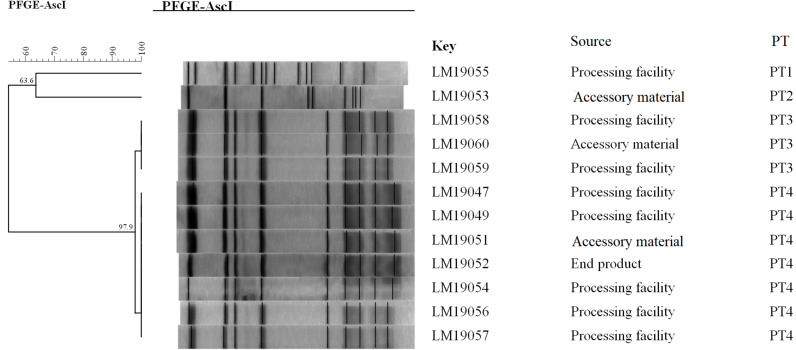
Relationships of the *L. monocytogenes* isolates based on PFGE. The 12 *L. monocytogenes* isolates were analyzed by PFGE using *Asc*I.

The PFGE analysis of the comprised *Asc*I divided the 12 isolates into 4 pulsotypes (PT1–4) (Figure 1). PT3 and PT4 values were similarly high (97.9%). They accounted for 83.3% (10/12) of the isolates. Two PTs (PT1, PT2) were presented by only one single isolate. Seven isolates in PT3 and PT4 were processing facility samples, two were from accessory material, and one was from an end product.

**FIGURE 2:**
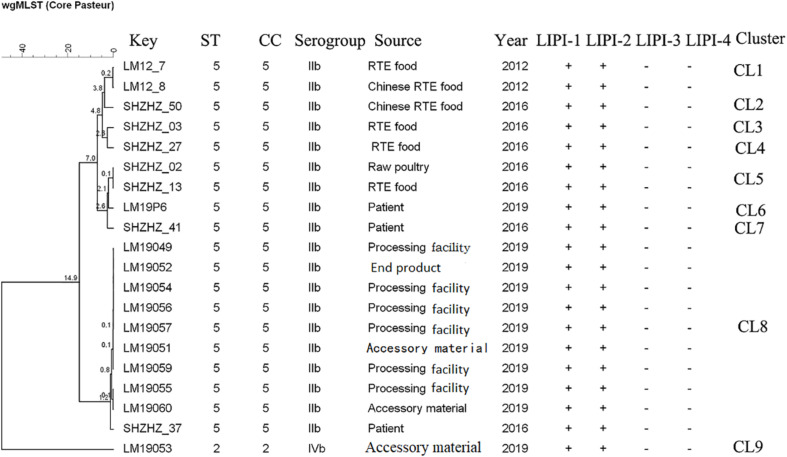
Minimum spanning tree of cgMLST data for 20 *L. monocytogenes* isolates. The multiplication by 10 in the tree represents the number of different alleles among isolates. The corresponding data, including the name of the isolate (key), MLST type (ST), MLST clonal complex (CC), source, year, *Listeria* pathogenicity island (LIPI-1, LIPI-2, LIPI-3, and LIPI-4), and cluster are shown alongside the dendrogram to the right.

The molecular typing using WGS data showed that 11 of 12 *L. monocytogenes* isolates belonged to ST5 (1/2b) with LIPI-1 (*Listeria* pathogenicity island-1). These ST5 isolates were compared with 10 other ST5 (1/2b) isolates from food products and patients using cgMLST. Nine clusters (CL1-CL9) were obtained (Figure 2). The *L. monocytogenes* isolates of three PTs (PT1, PT3 and PT4) and one clinical isolate belonged to CL8 and had 0–12 alleles based on cgMLST. However, >70 alleles were found between the isolates in CL8 and other CLs.

**FIGURE 3:**
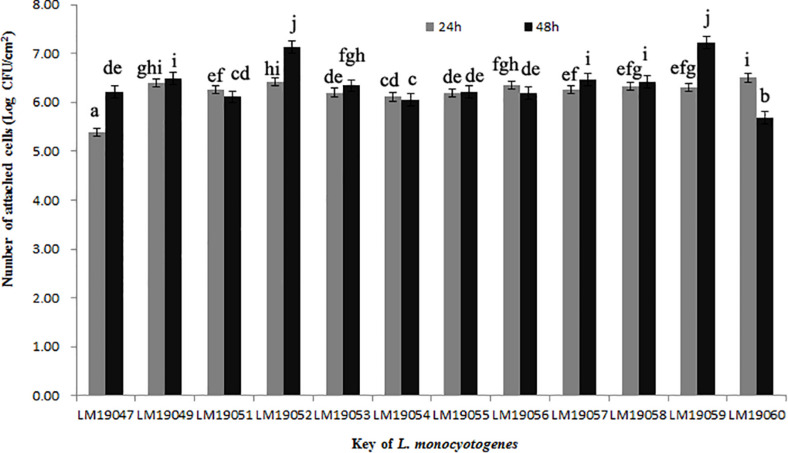
Population (Log CFU/cm^2^) of 12 *L. monocytogenes* isolates biofilms at 26°C on 24 and 48 h. Mean values at the different incubation time and different isolates with different lowercase letters are statistically different (*p* < 0.05).

After incubation at 26°C for 24 h, the minimum of attached cells of LM19047 isolates was 5.39 Log CFU/cm^2^, and the maximum of attached cells of 60 LM19060 isolates was 6.50 Log CFU/cm^2^, which were statistically different (*p* < 0.05) (Figure 3). However, with increasing biofilm age, the attached cells of LM19047 isolate increased up to 6.21 Log CFU/cm^2^, and that of LM19060 decreased to 5.69 Log CFU/cm^2^. The ability of biofilm forming of other isolates at different time was different (Figure 3).

## Discussion

Hygiene indicators (APC and coliforms) are used to reflect specific food establishment practices and temporal influences (Weatley et al., 2014). APC was confirmed in only one processing facility, while only one end product was confirmed with APC and coliform (Table 2). Furthermore, the mean level of APC in air samples in RTE processing rooms was 33 CFU/plate, which can be permitted in the food processing environment. These results indicated that good hygiene practices were implemented in this plant.

Contrarily, 8 of the 12 *L. monocytogenes* isolates were confirmed in 8 processing facilities, which presented the predominant contamination scenarios. These findings were similar to a previous study that was conducted on *L. monocytogenes* colonized under these scenarios in a meat establishment (Nastasijevic et al., 2017). The confirmation of *L. monocytogenes* was irrespective of the observed hygiene levels (for example, in contrast with the confirmation of *L. monocytogenes* in processing facilities, only one processing facility was confirmed with APC). Therefore, it was still difficult to remove *L. monocytogenes* isolates in this plant although good hygienic practices were implemented.

*L. monocytogenes* isolates were confirmed in accessory materials, intermediate products, and an end product, but not in raw materials, which suggested that *L. monocytogenes* isolates might be introduced to these products from processing facilities by cross-contamination. A similar contamination model reported that when *L. monocytogenes* entered into food processing plants, recontamination and persistence frequently occurred (Chambel et al., 2007). *L. monocytogenes* isolates were able to survive in niche areas of the facilities and could adapt to stress factors such as low temperature and low pH (Melo et al., 2015). Furthermore, *L. monocytogenes* isolates could persist in processing equipment for a long time (Unnerstad et al., 1996). As a result, contamination could exist for an extended period.

The epidemiological data showed that 12 *L. monocytogenes* isolates were detected in the same RTE meat processing plant at the same time, 10 of which shared an indistinguishable PT (PT3 and PT4, respectively) (Figure 1). Furthermore, the PFGE pattern of *L. monocytogenes* isolates in PT3 was highly similar to those in PT4, which might suggest the same ancestor. These results indicated clone transmission of *L. monocytogenes* isolates occurred in processing facilities in this plant.

Though PFGE is a useful tool for the characterization of the subtypes of *L. monocytogenes* isolates, it lacks discriminatory power to distinguish among closely related bacterial strains, which is essential for source tracking (Lomonaco and Nucera, 2014). Thus, WGS was used for obtaining better information about the genetic similarity between isolates. The indistinguishable *L. monocytogenes* isolates with the same PFGE pattern could be differentiated by cgMLST (Figure 2), which has a higher discriminatory power. cgMLST analysis of *L. monocytogenes* isolates in three PTs (PT1, PT2, and PT3) showed 0–8 allelic differences (Figure 2), these isolates could be identified as the same clone (Ruppitsch et al., 2015). Furthermore, seven of them were from processing facility samples, two from accessory materials, and one from an end product, which confirmed the clone transmission of *L. monocytogenes* isolates in the processing environment. Furthermore, in-house evolution occurred in *L. monocytogenes* isolates with one or eight alleles, which suggested that these *L. monocytogenes* isolates might have existed for a long period and also might be persistent in processing environments.

WGS data showed that most *L. monocytogenes* isolates (11/12) were ST5, CC5, 1/2b (Table 2). Our previous study indicated that the predominant *L. monocytogenes* isolates from both food and clinical isolates were ST5 in Shanghai, China (data unpublished). Similarly, ST5 was the most predominant ST in RTE meat product in Nanjing, China (Wang et al., 2015). Wang et al. reported that the ST5 had been identified as an important ST in China (Wang et al., 2012). There is no obvious difference between the distribution of the frequency of CC5 in foods and patients in France (Maury et al., 2016). The ST5 strains have been globally disseminated in geographically distant areas, e.g., Austria, Canada, Australia, Switzerland, and Finland (Schmid et al., 2014; Buchanan et al., 2017; Meier et al., 2017). Several outbreaks caused by *L. monocytogenes* have been linked to ST5 isolates (Buchanan et al., 2017). It is worth noting that 1 clinical isolate has 12 alleles with *L. monocytogenes* isolates obtained in this study belonging to CL8, which were from the RTE meat processing plant. Although there is a lack of epidemiological data confirming the relationship between them, the results suggested the potential risk of pathogenicity that these isolates pose to the consumers. Further studies are needed to uncover the pathogenicity of ST5.

ST5 isolates have been previously reported to be dominant in heavily contaminated food processing environments even after efforts on intensifying hygienic measures (Muhterem-Uyar et al., 2018). A further study indicated that ST5 plasmids harbored an efflux pump system (*bcrABC* cassette) and heavy metal resistance genes, which possibly provide a higher tolerance to disinfectants (Muhterem-Uyar et al., 2018). These *L. monocytogenes* isolates might exist in biofilm formation. *L. monocytogenes* biofilms could be formed on many different surfaces during food processing operations and provide a protective environment for bacterial survival and thereby increase the risk of subsequent contamination (Colagiorgi et al., 2017). Once established, *L. monocytogenes* biofilms act as permanent sources of contamination and dispersal in the environment and can lead to cross-contamination (Markkula et al., 2005). In this study, 12 *L. monocytogenes* isolates from the plant could form biofilm stainless steel coupons, which presented typical food-contact surfaces in food processing plants (Figure 3). These findings suggested that *L. monocytogenes* isolates in the processing equipment could not be cleaned due to biofilms formation, and *L. monocytogenes* biofilms might be persistent in the plant. Therefore, continuous surveillance and prevention strategies against *L. monocytogenes* should be implemented in this RTE meat processing plant to ensure food safety.

## Conclusion

In this study, cross-contamination of *L. monocytogenes* in an RTE meat plant has occurred. The food processing facilities were heavily contaminated by ST5 (1/2b) isolates even though good hygienic measures had been implemented in this plant. The molecular typing and epidemiological data confirmed that clone transmission occurred in the plant. Furthermore, the clone had a strong ability to form biofilm in food-contact surfaces, which might be the reason that it could not be eradicated in the processing facilities. Furthermore, the ST5 (1/2b) isolates in this study had potential pathogenicity for having 12 alleles with clinical isolate. Therefore, continuous surveillance and effective measures to eradicate *L. monocytogenes* should be taken to ensure food safety.

## Data Availability Statement

We have uploaded completely the WGS data of *L. monocytogenes* in this study in Genbank of NCBI.

## Author Contributions

HZ, FQ, and BX collected the samples, analyzed the samples, performed the WGS, and analyzed the data. HZ drafted the manuscript. XZ and HL designed the study and revised this manuscript. LS tested the formation of biofilm. YZ and WC performed the PFGE. YY was involved in the collection of isolates. QD revised this manuscript. All authors contributed to the article and approved the submitted version.

## Conflict of Interest

The authors declare that the research was conducted in the absence of any commercial or financial relationships that could be construed as a potential conflict of interest.

## Footnotes

1 http://www.mgc.ac.cn

2 http://bigsdb.pasteur.fr/listeria/listeria.html
